# Platelet lysate as a substitute for animal serum for the ex-vivo expansion of mesenchymal stem/stromal cells: present and future

**DOI:** 10.1186/s13287-016-0352-x

**Published:** 2016-07-13

**Authors:** Giuseppe Astori, Eliana Amati, Franco Bambi, Martina Bernardi, Katia Chieregato, Richard Schäfer, Sabrina Sella, Francesco Rodeghiero

**Affiliations:** Advanced Cellular Therapy Laboratory, Department of Cellular Therapy and Hematology, San Bortolo Hospital, Via Rodolfi 37, 36100 Vicenza, Italy; Transfusion Medicine and Cell Therapy, “A. Meyer” University Children’s Hospital, Florence, Italy; Hematology Project Foundation, Contrà S. Francesco 41, Vicenza, Italy; Department of Cell Therapeutics & Cell Processing, Institute for Transfusion Medicine and Immunohaematology, German Red Cross Blood Donor Service, Baden-Württemberg-Hessen gGmbH, Goethe-University Hospital, Sandhofstrasse 1, Frankfurt am Main, Germany

**Keywords:** Platelet lysate, Platelet releasate, Mesenchymal stem/stromal cells, Fetal bovine serum, Pathogen reduction, Manufacturing

## Abstract

The use of fetal bovine serum (FBS) as a cell culture supplement is discouraged by regulatory authorities to limit the risk of zoonoses and xenogeneic immune reactions in the transplanted host. Additionally, FBS production came under scrutiny due to animal welfare concerns. Platelet derivatives have been proposed as FBS substitutes for the ex-vivo expansion of mesenchymal stem/stromal cells (MSCs) since platelet-derived growth factors can promote MSC ex-vivo expansion. Platelet-derived growth factors are present in platelet lysate (PL) obtained after repeated freezing–thawing cycles of the platelet-rich plasma or by applying physiological stimuli such as thrombin or CaCl_2_.

PL-expanded MSCs have been used already in the clinic, taking advantage of their faster proliferation compared with FBS-expanded preparations. Should PL be applied to other biopharmaceutical products, its demand is likely to increase dramatically. The use of fresh platelet units for the production of PL raises concerns due to limited availability of platelet donors. Expired units might represent an alternative, but further data are needed to define safety, including pathogen reduction, and functionality of the obtained PL. In addition, relevant questions concerning the definition of PL release criteria, including concentration ranges of specific growth factors in PL batches for various clinical indications, also need to be addressed. We are still far from a common definition of PL and standardized PL manufacture due to our limited knowledge of the mechanisms that mediate PL-promoting cell growth. Here, we concisely discuss aspects of PL as MSC culture supplement as a preliminary step towards an agreed definition of the required characteristics of PL for the requirements of manufacturers and users.

## Background

Mesenchymal stem/stromal cells (MSCs) are defined as self-renewing, multipotent progenitor cells able to differentiate into other cell types of mesodermal origin, such as adipocytes, osteocytes, and chondrocytes [1].

MSCs isolated from culture medium show consistent phenotypic characteristics such as adherence to plastic surfaces, positivity for cell-surface molecules CD105, CD73, and CD90, and negativity for hematopoietic markers and HLA-DR [2]. As well as this shared marker profile, different MSC subpopulations may feature phenotypical and functional heterogeneity [3, 4].

MSCs exert potent immunosuppressive and anti-inflammatory activities [5] by suppressing T-cell proliferation in vitro [6–8], via direct cell-to-cell contact [9] and by the production of soluble factors, including nitric oxide [10], hepatocyte growth factor (HGF) and transforming growth factor (TGF)-β1 [8], and indoleamine 2,3-dioxygenase (IDO) [11].

The use of fetal bovine serum (FBS) and other animal derivatives for the ex-vivo expansion of MSCs has been discouraged by regulatory authorities [12–14] to reduce the risk of transmitting prions and other zoonoses and to avoid xenogeneic immune reactions in the host. However, the lack of standardization of FBS preparations leads to inconsistency in cell culture performance [15] and FBS production has come under scrutiny because of animal welfare concerns [16, 17].

Platelet lysate (PL) was initially proposed as an alternative to animal serum for the ex-vivo expansion of MSCs by Doucet et al. [18]. Bioactive molecules and growth factors contained in PL support the expansion of MSCs derived from bone marrow (BM) [19–22], umbilical cord blood (UCB) [23, 24], and adipose tissue (AT) [25–27], showing favorable results compared with FBS. In addition, MSCs expanded with PL-enriched medium have been used for the treatment of patients with steroid-refractory acute graft versus host disease (GVHD) [28–33] after hematopoietic stem cell transplantation and of patients suffering from several orthopedic disorders, mainly moderate to severe osteoarthritis of the knee [34].

The isolation and expansion of MSCs in xeno-free conditions using PL could thus represent a valuable alternative to FBS.

## Review

### Production and use of animal serum in cell cultures

Animal serum, used historically for culturing cells [35], is a composite combination of biomolecules with different growth-promoting and inhibiting activities. Its major function in culture media is to deliver trophic factors stimulating cell proliferation and to provide transport proteins, minerals, trace elements, lipids, attachment factors, and stabilizing and detoxifying elements needed for maintaining pH or to inhibit proteases and other toxic molecules [36]. FBS is obtained in slaughterhouses from fetuses of healthy dams destined for human consumption. FBS is superior to serum from adult animals because of its reduced γ-globulin content, thereby reducing the risk of possible antibody interactions with cell cultures.

In this context, a case of fraud came up in 2013 [37] when it was discovered that some lots of FBS produced between 2003 and 2011 were subject to label nonconformances [38].

In the last 10 years substantial efforts have been made to identify substitutes of animal serum, including serum-free media [19, 39] and byproducts obtained from the lysis or the activation of human platelets. However, the use of alternative media still remains largely unexplored and animal serum is still an essential component, for instance, in the production of vaccines.

### Human PL preparation

**Fig. 1:**
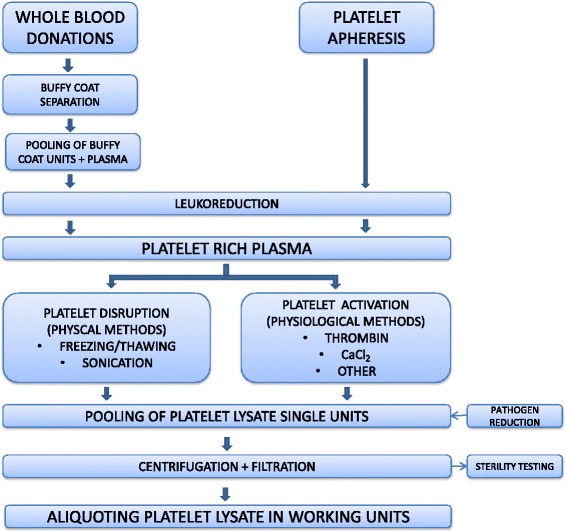
Procedure for PL and platelet releasate preparation

Release of growth factors from platelets can be achieved by repeated freezing–thawing cycles of the platelet-rich plasma (PRP) obtained from platelet apheresis or from the buffy coat (Fig. 1). Briefly, the PRP bags are frozen overnight at –80 °C and then thawed at +37 °C; this cycle is repeated one to three times. After pooling and one or more centrifugation/filtration steps in order to remove cellular debris, PL is ready to be added to the growth media (reviewed in [40]). Preparation procedures adopted in different laboratories may vary with regard to the use of fresh or expired platelets, the number of freezing–thawing cycles, pathogen reduction (PR), and filtration steps, causing variations in the concentration and integrity of the growth factors released that are likely related to the efficacy of the platelet granules’ disruption [18].

Sonication to produce PL has been described previously by Hara et al. [41] alone or in combination with a freezing–thawing cycle [42]. Ultrasounds are sounds having a frequency ≥20 kHz. Their effect is based on the transmission of ultrasounds in a liquid where they generate thermal and nonthermal effects. For the latter, ultrasound waves act on the gas dissolved, where the compression of the liquid is followed by its rarefaction. As a consequence, the micro bubbles expand with each cycle of the applied ultrasonic energy until they reach an unstable size, and then they collide and/or violently collapse in a process called “cavitation” [43, 44].

We have described previously the production of PL from fresh PRP using ultrasounds at a frequency of 20 kHz [45]. The efficiency of the lysis was estimated after quantification of the platelet-derived growth factor-AB (PDGF-AB). After 30 minutes of sonication, 74 % of PDGF-AB was released from the platelet granules in the medium.

Platelet factors can be also released by physiological stimuli as thrombin, collagen, adenosine diphosphate, epinephrine, and thrombin receptor-activating peptide [46], CaCl_2_ [47], or tri-*n*-butyl phosphate and Triton X-45 [48] in order to obtain the so-called platelet releasate (reviewed in [49]). The methods used to release the platelet factors are an important variable leading to lot-to-lot variations of the final product, but few studies have addressed this topic.

It has been observed that PL obtained after platelet activation with thrombin (platelet releasate) and PL obtained by platelet freezing/thawing stimulate different proliferation rates of BM-derived MSCs [20]. In particular, the platelet releasate significantly accelerated BM-MSC proliferation to yield cell numbers clinically relevant within the first two passages. MSC quality and functionality including cell surface marker expression, adipogenic and osteogenic differentiation, and immunosuppressive action were similar in MSCs from all culture conditions.

We expanded BM-MSCs in medium containing 10 % platelet releasate produced by CaCl_2_ activation, observing that the cumulative population doubling time of cells at passage 8 was about twice that of the same cells expanded in medium containing 10 % PL obtained by repeated freezing–thawing cycles. We did not observe any differences in phenotype and immunomodulation properties of the cells expanded in the two conditions [50]. Also, previous studies reported that repeated freezing and thawing cycles have rather negative effects on growth factor content [51, 52]. Further studies are needed to investigate the possible effects of different PL manufacturing technologies on cell expansion, differentiation, and immunomodulation.

### Use of expired platelet units for PL production

The use of fresh platelet units for the production of PL raises concerns due to the limited availability of donors in the context of demand for platelet units in the clinic. Expired platelet units may represent an alternative source since there is evidence that platelets obtained from expired units can be used without compromising the quality of the final product [53].

In an Italian region of 3.8 million inhabitants, about 100,000 buffy coat units from single donors were produced in 2014. Of these, about 60 % were discarded after the expiration date. Considering a mean buffy coat volume of 50 ml, about 3000 L of PL could have been produced using only the expired buffy coat units.

### Platelet-derived factors

Platelets contain bioactive molecules and growth factors that are released from α-granules after platelet destruction by physical or physiological methods. Among these are coagulation factors, adhesion molecules, protease inhibitors and proteoglycans, basic fibroblast-derived growth factor (bFGF), epidermal growth factor (EGF), HGF, vascular endothelial growth factor (VEGF), insulin-like growth factor-1 (IGF-1), TGF-β1, soluble CD40L, vascular cell adhesion molecule-1, intercellular adhesion molecule-1, PDGF-AA, PDGF-AB, PDGF-BB, chemokine (C-C) ligand 5, and chemokine (C-X-C) ligand 1/2/3 [54, 55]. All of these molecules influence cell proliferation and function [56] and could promote proliferation in comparison with FBS [20, 57].

The role of these factors in cell expansion is only partially understood. Neutralization experiments indicated that some platelet factors are essential for MSC proliferation, and thus inhibition of PDGF-BB and bFGF decreases MSC proliferation by about 20 % and 50 %, respectively [55].

Extensive functional and differential proteomic analysis to identify platelet-derived factors affecting ex-vivo expansion of MSCs was performed by Kinzebach et al. [58] using MALDI-TOF and western blotting and by Horn et al. [59] using human cytokine antibody arrays. In Horn et al.’s study, the chemokine profiles of eight PLs were correlated with proliferation activity showing a significant positive effect of increasing concentrations of PDGF-AB.

The content of platelet factors could be different for platelet releasate produced from cord blood (CB) or peripheral blood (PB) [60]. Using a wide proteomic array, the authors discovered that several hormones strongly supporting fetal tissue formation like prolactin, progesterone, and α-fetoprotein were present only in platelet releasate obtained from CB. Moreover, in CB releasate the authors identified higher concentrations of factors known to promote angiogenesis, such as VEGF. On the contrary, the proteomic analysis showed that platelet releasate obtained from PB contained more proinflammatory factors such as chemokine CC4, metalloproteinase 3, and chemokine (C-C motif) ligand 5.

Using high-throughput proteomic array analysis, an accurate proteomic dissection of PL was performed by Crespo-Diaz et al. [61]. Within the extracellular signaling molecules of PL, FGF/EGF, TGF-β/bone morphogenic protein (BMP), and VEGF/PDGF were highly represented.

### Isolation and ex-vivo expansion of MSCs in PL-enriched medium

In their seminal study Doucet et al. [18] expanded MSCs in FCS or in a medium supplemented with PL, demonstrating that the latter promoted MSC expansion and thus decreased the time required to reach confluence while increasing the fibroblastoid colony-forming unit (CFU-F) size when compared with FCS cultures. MSCs cultured in the presence of PL maintained their trilineage differentiation potential and their immunosuppressive activity.

Schallmoser et al. [62] provided evidence that PL could replace FBS for clinical-scale expansion of MSCs. PL was more efficient than FBS in supporting MSC expansion and, although morphologically distinct, MSCs did not differ significantly in terms of immunophenotype, differentiation potential, and lack of tumorigenicity in mice.

Capelli et al. [63] demonstrated that PL allowed clinical-grade production of MSCs starting from diagnostic samples of BM aspirates or using the bag and filter remnants at the end of BM infusions. A significantly faster expansion was obtained with PL, compared with FBS. No differences were observed in terms of morphology, differentiation potential, surface markers, and immunological properties. The same authors demonstrated that umbilical cord derived-MSCs can also be expanded in PL [64].

### Alterations of the immune-regulatory effect of MSCs expanded in PL

PL may alter the expression of some relevant MSC surface molecules, impairing their inhibitory capacity on T-cell proliferation to alloantigen and NK-cell proliferation and cytotoxicity [65]. Diminished immunosuppressive properties for both resting and interferon-γ-primed BM-MSCs and AT-MSCs was also reported [66], together with attenuated expression levels of IDO-1 compared with FBS.

On the contrary, our group [57] demonstrated a stronger inhibitory effect on lymphocyte proliferation with AT-MSCs expanded in PL when compared with AT-MSCs expanded in FBS or in human platelet-poor plasma (hPPP). Moreover, Flemming et al. [67] evidenced that BM-MSCs expanded in PL had comparable inhibitory effect on lymphocyte proliferation compared with their FCS cultured counterparts. The data were further confirmed by Bernardo et al. [68].

### PL biosafety

The risk of cell transformation during MSC ex-vivo expansion was addressed by Crespo-Diaz et al. [61] by evaluating chromosomal stability of BM-MSCs after long-term culture in PL or FBS. Notably, no clonal karyotypic abnormalities were observed at passage 12.

Our group investigated whether the increased MSC proliferation achieved with PL could induce chromosomal instability. Reassuringly, as showed by our group [69], micronuclei formation in Chinese Hamster Ovarian K1 cell lines exposed to increasing concentrations of PL was unchanged. The senescence of MSCs cultured for up to 16 passages in medium containing FBS or PL was assessed by endogenous β-galactosidase expression [70]. MSCs cultured with FBS for different numbers of passages were switched to PL conditions to evaluate the ability of PL to “rescue” the proliferative capacity of MSCs. Interestingly, PL culture of aged and senescent MSCs demonstrated cellular rejuvenation, reflected by decreased doubling time and smaller cell size. At this point, the mechanisms behind this observation have not been elucidated—but it may be speculated that a specific growth factor such as EGF or, more likely, a combination of growth factors in the PL may mediate its beneficial effects on aged MSC cultures.

Gene expression changes after FBS or PL culture were investigated by Schallmoser et al. [71]. Surprisingly, all BM-MSC preparations revealed significant gene expression changes upon long-term culture; in particular, genes involved in cell differentiation apoptosis and cell death were upregulated whereas genes involved in mitosis and proliferation were downregulated, indicating that long-term expansion induced similar gene expression changes in BM-MSCs irrespective of isolation and expansion conditions.

Lohmann et al. [72] analyzed the impact of the donor age on PL functionality, observing that MSC proliferation was significantly higher in PL derived from younger donors, and PL from older donors increased activity of senescence-associated β-galactosidase.

### Pathogen reduction

#### Strategies for pathogen reduction

In addition to viral contamination, platelet units are at particular risk of bacterial contamination by adventitious pathogens at the site of venipuncture or bacteremia of donors. PR can therefore be implemented in the manufacturing process to lower bacterial and viral loads [73–75]. Current PR technologies comprise solvent detergent treatment, methylene blue/light, riboflavin/ultraviolet light, or amotosalen/ultraviolet light (Intercept™) [76].

Although PR can likely reduce the transmission risk of known and as yet unknown infectious diseases, recent studies reporting on the effects of PR on transcriptomes and proteomes of platelets [77, 78] highlight the need for further studies to evaluate the effect of PR in the manufacturing process of PL.

Shih et al. [79] compared FBS with inactivated PL for expansion of AT-MSCs, concluding that the treatment did not alter the differentiation capacity or the cell immunophenotype.

Systems for pathogen inactivation of platelet donations have been developed based on the disruption of nucleic acids by a photoactivation process using psoralens plus ultraviolet light, and the PL produced with inactivated donations was able to sustain BM-MSC expansion and immunoregulation [80, 81].

The functionality of PL prepared starting from expired units treated and untreated with a PR system (Intercept™) was also tested by evaluating immunomodulation, immunophenotype, proliferation, and trilineage differentiation of MSCs. Interestingly, the conclusion was that PL prepared from expired and pathogen-reduced platelets supported MSC differentiation and immunosuppression better than untreated PL [82].

## Conclusion

A substantial quantity of laboratory data on the preparation modalities and on the characterization of PL have been produced, suggesting that MSCs expanded in PL grow faster when compared with MSCs expanded in FBS-enriched media. If the use of PL could be escalated to the manufacturing of other cell therapeutics or biopharmaceutical products, the demand for PL is likely to increase dramatically. In this future scenario, several questions should be addressed.

### Supply

The annual FBS availability is estimated to be around 500,000–600,000 L/year [36, 83], of which about 1/3 is suitable for Good Manufacturing Practices (GMP) production. In recent years, the annual demand of serum has decreased mainly because the production of vaccines has switched to the use of serum-free microbial or mammalian cell cultures [84]. In parallel, animal serum production and availability has decreased worldwide because of a diminished demand from the vaccine industry and the huge number of cattle reared for beef and dairy [84], thus limiting the accessibility of this product. The decline in request and production of bovine serum has occurred in parallel with increasing demand for cell therapeutics and regenerative medicine products. The worldwide availability of blood donations therefore needs to cover clinical demand, cell manufacture, and research [40]. In this context, expired platelet units may emerge as the main source for PL, even with the lack of agreed quality criteria. The process of PL production, characterization, and testing, mainly driven by academia and increasingly blood banks, could hardly compete with industrial manufacturers that now offer PL produced under GMP.

### Processing

Several methods have been proposed to release growth factors from platelets resulting in different release efficiency and PL efficacy. A consensus should be obtained on the standardization of the method(s) used.

### Release criteria

Even if some studies suggest a major role for some specific cytokines in PL, a consensus has not so far been found. One question is whether or not the same cytokine content is critical for different cell types. In this sense, one of the fundamental steps in PL definition would be the designation of release criteria.

The PL or releasate production method seems to play a role in the final composition of the product, possibly modulating the quantity and quality of factors released from platelet granules. The final concentration of cytokines and growth factors in PL is also likely linked to the number of platelet units pooled and the final platelet concentration.

Since PL contains both plasma and platelet proteomes, the concentration of human immunoglobulin G (IgG) in a preparation could vary between 8 and 12 mg/ml [40] and could also be related to the production process and the plasma concentration in the final product. In order to reduce the risk of side effects in the patient caused by a high concentration of allogeneic IgG in the cell preparation, the IgG concentration in the PL preparation should be defined.

Before releasing the product, the ability to expand a reference MSC preparation to a predefined level at least comparable with FBS might be a measure to assess PL quality. The immunomodulatory properties of MSCs expanded in PL or releasate should be maintained and it would be favorable to develop a standard assay to evaluate this function. Release criteria should also include the endotoxin content and viral and bacterial safety.

In conclusion, we have identified substantial need to define PL and to understand the mechanisms that mediate the beneficial effects of PL on cell growth. Collaborative efforts of scientific societies, end users, blood centers, and industry are urgently needed to improve our knowledge.

## Abbreviations

AT, adipose tissue; bFGF, basic fibroblast-derived growth factor; BM, bone marrow; BMP, bone morphogenic protein; CB, cord blood; CFU-F, fibroblastoid colony-forming unit; EGF, epidermal growth factor; FBS, fetal bovine serum; GMP, Good Manufacturing Practices; GVHD, graft versus host disease; HGF, hepatocyte growth factor; hPPP, human platelet-poor plasma; IDO, indoleamine 2,3-dioxygenase; IGF-1, insulin-like growth factor-1; IgG, Human immunoglobulin G; MSC, mesenchymal stem/stromal cell; PB, peripheral blood; PDGF, platelet-derived growth factor; PL, platelet lysate; PR, pathogen reduction; PRP, platelet-rich plasma; TGF, transforming growth factor; UCB, umbilical cord blood; VEGF, vascular endothelial growth factor
